# Supplementary material for: Identification of photoperiod-induced specific miRNAs in the adrenal glands of Sunite sheep (Ovis aries)
Source: Front Vet Sci. 2022 Jul 22;9:888207. doi: 10.3389/fvets.2022.888207 (PMC9354845; doi:10.3389/fvets.2022.888207)
Supplement: Supplementary file 1 [file Data_Sheet_1.ZIP › Supplementary file/miRDeep2 file.zip!/miRDeep2 file/Novel_Structure/LP42A1/Novel_263.pdf]

| | | |
|---|---|---|
| Provisional ID | : | NC_019469.2_20635 |
| Score total | : | 21.7 |
| Score for star read(s) | : | 3.9 |
| Score for read counts | : | 14.4 |
| Score for mfe | : | 1.8 |
| Score for randfold | : | 1.6 |
| Score for cons. seed | : | |
| Total read count | : | 40 |
| Mature read count | : | 10 |
| Loop read count | : | 0 |
| Star read count | : | 30 |

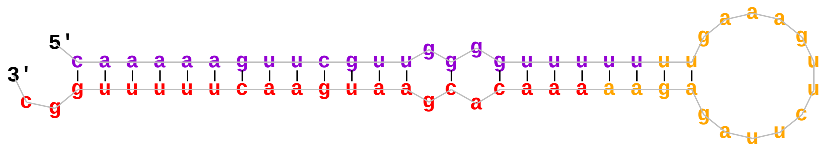


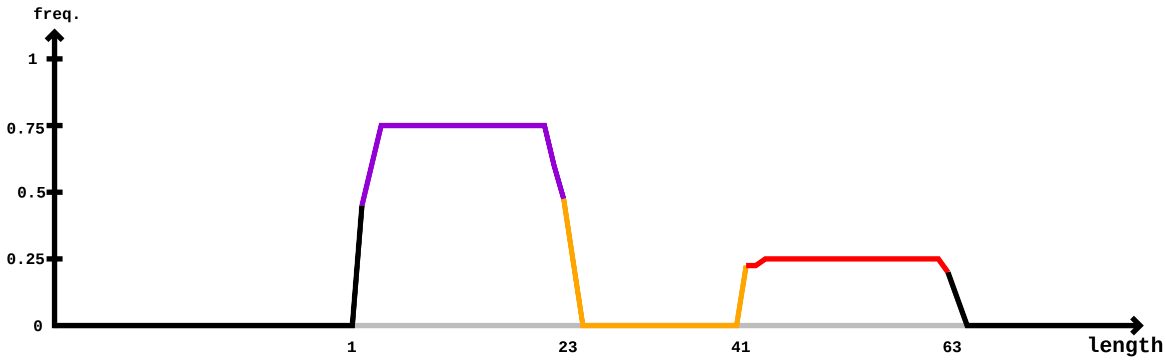


```
                                               Star                                      Mature
5'- gcuuggguggcaccaaaauauugggauggccaaaaaguucguugggguuuuuuugaaaguucuuagagaaaaacacgaaugaacuuuuuggcuaguccuaaagaccccuga   -3'    obs
    gcuuggguggcaccaaaauauugggauggccaaaaaguucguugggguuuuuuugaaaguucuuagagaaaaacacgaaugaacuuuuuggcuaguccuaaagaccccuga          exp
    ..((((......))))....(((((((((((((((((((((((.(.((((((((............)))))))).).))))))))))))))))).))))))..........   reads   mm   sample
    ..............................caaaaaguucguuUggguuuu............................................................   5       1    seq
    ..............................caaaaaguucguuCggguuuuu...........................................................   6       1    seq
    ..............................caaaaaguucguuUggguuuuu...........................................................   7       1    seq
    ...............................aaaaaguucguuUggguuu.............................................................   6       1    seq
    ................................aaaaguucguuUggguuuuuu..........................................................   6       1    seq
    ......................................................................aaacacgaaugaacuuuuugg....................   2       0    seq
    ......................................................................aaacCcgaaugaacuuuuuggc...................   7       1    seq
    ........................................................................acCcgaaugaacuuuuuggcu..................   1       1    seq
```
